# Skin Deep: An Overlap of Delusions

**DOI:** 10.7759/cureus.62681

**Published:** 2024-06-19

**Authors:** Khulood Abdulraouf Almarzooqi, Dimitre Dimitrov, Khaled Alharmoodi

**Affiliations:** 1 Department of Dermatology, Sheikh Khalifa Medical City, Abu Dhabi, ARE; 2 Department of Psychiatry, Essex Partnership University NHS Foundation Trust, Colchester, GBR

**Keywords:** delusional infestation, morgellons disease, ekbom syndrome, delusions of parasitosis, schizophrenia, scabies, delusion of infestation

## Abstract

Delusional infestation (DI) describes a fixed, false belief where a person believes that they are infested with living or inanimate pathogens despite the absence of medical evidence for such infestation. Descriptions of alleged pathogens have evolved over time, incorporating inanimate objects such as fibrous strands. With the emergence of Morgellons disease and its controversy, we report a case of a 40-year-old female presenting with a strong belief of scabies infestation along with fibers emerging from her skin. Further, although insects are still the most alleged source of infestation, the overlap of Morgellons disease and the delusion of infestation supports it as a DI variant and questions the notion of its existence as a separate diagnostic entity.

## Introduction

Delusion of infestation, formerly recognized as delusional parasitosis or Ekbom syndrome, is a monosymptomatic hypochondriacal psychosis in which affected individuals have a fixed belief that their body or immediate environment is infested by small, living (or less often inanimate) pathogens despite the lack of any medical evidence for this [1]. Delusional and psychotic disorders have stable main delusional themes, but their specific content can vary due to external influences. In the past, patients believed themselves to be infested by scabies, typhus, or pests. This changed even more recently, with the alleged pathogens being threads, hairs, and fibers, a phenomenon that afflicted persons call Morgellons disease.

Morgellons disease is characterized by the perception of fibers or threads emerging from or attached to the skin [1]. It is a subtype of delusional infestation (DI) and is further supported by the high level of coexisting psychiatric conditions; the most common associated diagnoses were depression, substance abuse, and anxiety [2]. Delusional infestation can present as the classic form, primary delusional infestation, which develops without any known cause or underlying illness and meets the criteria for a persistent delusional disorder or, in the context of other medical or psychiatric diseases, as a secondary diagnostic entity [1].

Patients with DI can often be readily identified. However, akin to other delusional disorders, delusional parasitosis is considered challenging due to the necessity of excluding a multitude of potentially associated illnesses and evaluating their causative role.

## Case presentation

A 40-year-old Iraqi female presented to our clinic with a four-year history of a firm belief that her body was infested by scabies. She reported that her symptoms began following a visit to the beach, during which she thought scabies entered her skin through her feet. The patient disclosed distressing symptoms related to her delusional belief including seeing and feeling the presence of scabies crawling under her skin. She also described the sensation of scabies inside her mouth, which she believed had caused her teeth to fall out. Her fear extended to concerns that these infestations could potentially reach her brain. Additionally, the patient noted the presence of threadlike fibers emerging from her skin. Gradually, it impaired her quality of life and sleep, leading to the loss of her job and avoiding sleeping on any mattress from fear of infestation. There was difficulty initiating and maintaining sleep because of the unpleasant sensations. Desperate to eliminate the infestation, she would discard all her clothing permanently after wearing them.

Her past medical history did not reveal any significant medical conditions or relevant issues that could explain her current distressing symptoms. She also denied taking any new medications or drug use. In her social history, the patient mentioned a significant life event that she believes contributed to her deteriorating mental health. In 2020, she experienced the loss of her father, an event she identified as a significant trigger for her emotional and psychological decline. She currently resides in Jordan alone, and her relationship with her family is described as distant and lacking in emotional closeness. She denied any suicidal thoughts or plans, behavior of self-harm, or any episodes of overt manic symptoms.

**Figure 1:**
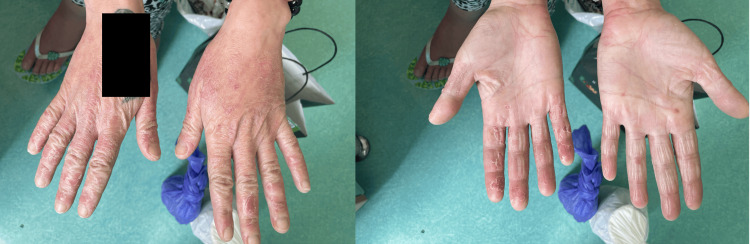
Bilateral eczematous patches were seen on the dorsum and palms of the hands secondary to the use of bleach.

**Figure 2:**
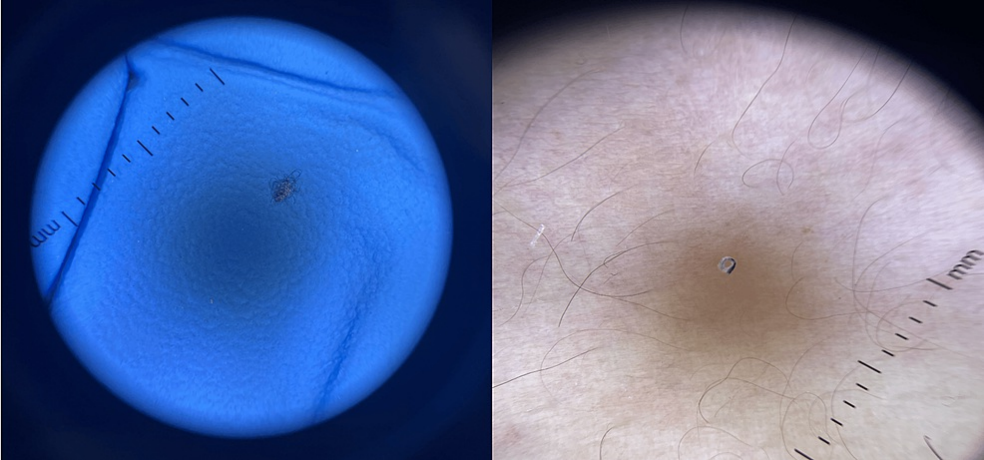
Fibers and threads were seen on the patient's skin with a dermatoscope.

Throughout her medical history, the patient received multiple courses of permethrin from various dermatologists and claimed a diagnosis of Norwegian scabies. She also bathed herself with bleach daily with no improvement. Upon examination, multiple excoriated papules with hemorrhagic crusts, as well as postinflammatory hyperpigmentation and scarring, were noted on her back and extremities. Other findings included bilateral lower limb edema. Additionally, eczematous patches were seen bilaterally on the dorsum and palms of the hands, secondary to using bleach, as seen in Figure 1. The patient also pointed to fibers on her skin during the examination, as shown in Figure 2.

**Figure 3:**
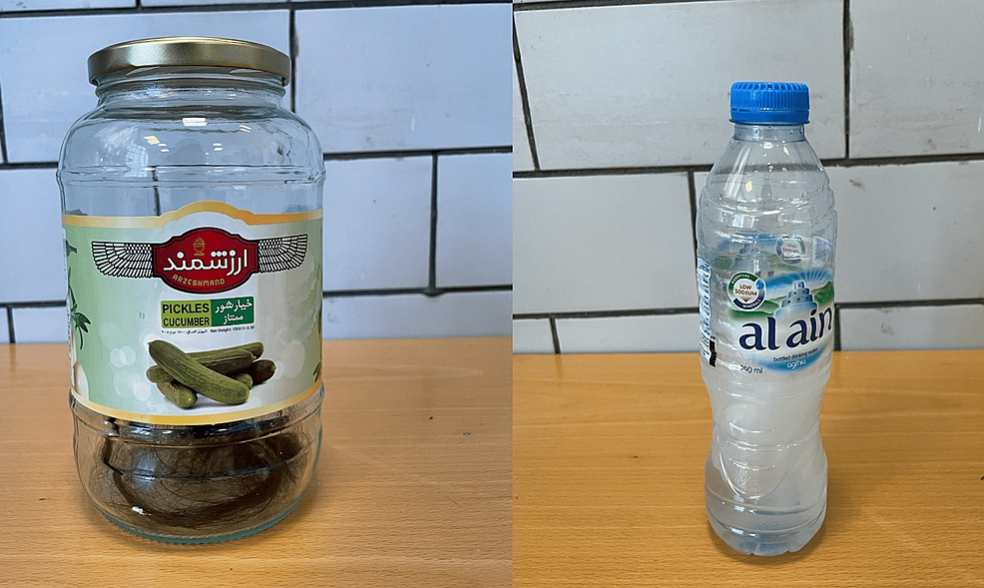
Specimens displayed include a hair extension contained in a pickle jar and scales collected in a water bottle.

Further, a specimen of hair extension in a pickle jar and a water bottle with scales were provided for examination, as seen in Figure 3. During the mental status examination, her mood was consistently anxious, and while she appeared distressed, her affect remained stable. Her thought content included delusions of scabies infestation, along with a belief in fibers emerging from her skin. She reported both visual and tactile hallucinations related to scabies. Additionally, the patient lacked insight into her condition.

Comprehensive investigations, including routine laboratory investigations, C-reactive protein, thyroid function tests, and liver and renal function tests, were all within normal ranges. The electrocardiogram and urine toxicology screen were also clear. Skin scraping did not reveal any mites or scybala under the microscope. After a comprehensive assessment, the treatment plan was discussed with the patient. Upon the prescription of the antipsychotic risperidone, the patient revealed that her mother takes the same medication and has a history of delusional disorder due to an ongoing belief of having different cancers. Subsequently, she was not convinced about the treatment, ultimately losing follow-up.

## Discussion

The patient's presentation is characterized by the convergence of dermatological and psychiatric manifestations, introducing complexities in the diagnostic and therapeutic processes. In clinical practice, it is generally observed that specific demographic profiles are more commonly associated with DI. Among these, the most prevalent demographic consists of middle-aged to elderly females exhibiting limited social interactions and no history of psychiatric disorders and possessing normal cognitive and social capabilities [3]. Furthermore, no discernible correlation exists with socioeconomic status, education, or childhood difficulties. Research has also been unable to identify any specific personality traits [1].

The specific etiology of DI remains elusive and is considered to be multifaceted. Nonetheless, certain delusional and psychotic states have been correlated with structural brain abnormalities and dopaminergic neurotransmission irregularities. One proposed mechanism involves a potential increase in extracellular dopamine levels within the brain's striatum, likely due to a reduced functionality of dopamine transporters that facilitate the neurotransmitter's transport [4]. Moreover, MRI studies of DI patients have shown decreased brain volume in areas that regulate probabilistic reasoning and body perception [5].

According to the Diagnostic and Statistical Manual of Mental Disorders, Fifth Edition (DSM-5), it is classified as a delusional disorder, somatic subtype, with the delusion of infestation as the sole psychotic symptom. The symptoms should be present for a duration of one month or longer and should not be attributable to another disorder, substance, or medication effect [6]. The symptoms should not meet criterion A for schizophrenia, and any hallucinations present should not be prominent and should be related to the delusional theme [6].

There is no uniformity in the presentations of individual patients with DI. Patients may describe tactile, visual, or auditory hallucinations related to the infestation [7]. Onset is insidious, and patients would usually have multiple visits to health professionals with symptoms being present for months to years before diagnosis [8]. The mean duration of the disease is approximately three years [2]. In our case, disclosing a positive family history posed a challenge. In a published study, a positive psychiatric family history was reported in 15% of DI cases; this rate did not differ from that for schizophrenia (16%), but both were higher than those for control families [9].

Patients frequently embark on a journey influenced by their belief in the delusion, resulting in a substantial investment of time, finances, and energy to eradicate these perceived insects. It is noteworthy that approximately 90% of individuals experiencing delusional infestation (DI) initially seek assistance from dermatologists [10]. Despite unremarkable previous tests, many patients seek a more comprehensive or definitive diagnostic workup. The examination of the patient and specimens is instrumental in establishing a trusting relationship, which forms the basis for subsequent treatment.

Successful engagement in treatment necessitates the establishment of trust, wherein the clinician aligns with the patient without endorsing the delusion. Healthcare providers are advised to inform patients that their symptoms may result from overactivity in the nervous system, aiming to enhance compliance with medication use [5]. Patients often require reassurance that antipsychotic medications are not being prescribed for treating schizophrenia or other psychotic disorders, as this can deter them from seeking treatment [11]. Based on published data on antipsychotic efficacy, the incidence of side effects, and attributable risk, risperidone (0.5-4 mg/day) is a reasonable first-line choice for pharmacotherapy [6]. Adverse effects are generally tolerable and infrequent at low doses, primarily manifesting as mild anticholinergic effects. However, higher doses are associated with effects attributed to dopamine blockade, such as weight gain, galactorrhea, and extrapyramidal symptoms [5].

The onset of a clinical response typically occurs within 1.5-2 weeks following the initiation of pharmacotherapy, with the maximum effect frequently achieved within six weeks [5]. It is imperative to recognize that the discontinuation of therapy after the attainment of a clinical response often precipitates symptom relapse [6]. In addition to pharmacological interventions, cognitive behavioral therapy (CBT) is a viable treatment modality as it can help patients question their fixed beliefs, cease obsessive behaviors, and connect their thoughts, emotions, and behaviors [12]. Further, by focusing on re-evaluating fearful thoughts of the infestation, CBT can help build an alliance with the patient, lessen their anxiety, and improve their social functioning [13].

## Conclusions

Dermatologists should collaborate with psychiatrists to address these conditions' physical and psychological aspects. Accurate and timely diagnosis is crucial to relieve the burden faced by patients through a patient-centered and sensitive approach. Furthermore, while insects persist as the predominant alleged source of infestation, the coexistence of Morgellons disease and delusional infestation underscores the latter as a variant of delusional infestation, thereby challenging the conceptualization of Morgellons disease as a distinct diagnostic entity.
